# Genetic analyses and dispersal patterns unveil the Amazonian origin of guava domestication

**DOI:** 10.1038/s41598-024-66495-y

**Published:** 2024-07-08

**Authors:** Edna Arévalo-Marín, Alejandro Casas, Hernán Alvarado-Sizzo, Eduardo Ruiz-Sanchez, Gabriela Castellanos-Morales, Lev Jardón-Barbolla, Gustavo Fermin, José S. Padilla-Ramírez, Charles R. Clement

**Affiliations:** 1 Instituto de Investigaciones en Ecosistemas y Sustentabilidad-IIES, Universidad Nacional Autónoma de México, Morelia, Michoacán Mexico; 2 Posgrado en Ciencias Biológicas, Unidad de Posgrado, Ciudad Universitaria, Coyoacán, CDMX Mexico; 3 Laboratorio de Biogeografía y Sistemática, Departamento de Biología Evolutiva, Facultad de Ciencias, Universidad Nacional Autónoma de México, Ciudad Universitaria, Mexico City, CDMX Mexico; 4 Departamento de Botánica y Zoología, Centro Universitario de Ciencias Biológicas y Agropecuarias, Universidad de Guadalajara, Zapopan, Jalisco Mexico; 5 Departamento de Conservación de la Biodiversidad, El Colegio de la Frontera Sur, Unidad Villahermosa (ECOSUR-Villahermosa), Villahermosa, Tabasco Mexico; 6 Centro de Investigaciones Interdisciplinarias en Ciencias y Humanidades, Universidad Nacional Autónoma de México, Ciudad Universitaria, Coyoacán, CDMX Mexico; 7 Instituto Jardín Botánico de Mérida, Facultad de Ciencias, Universidad de Los Andes, Mérida, Mérida Venezuela; 8 Campo Experimental Pabellón, Instituto Nacional de Investigaciones Forestales, Agrícolas y Pecuarias (INIFAP), Aguascalientes, Mexico; 9 Instituto Nacional de Pesquisas da Amazônia, Manaus, Amazonas Brazil

**Keywords:** Plant domestication, Plant genetics, Population genetics

## Abstract

Guava (*Psidium guajava* L.) is a semi-domesticated fruit tree of moderate importance in the Neotropics, utilized for millennia due to its nutritional and medicinal benefits, but its origin of domestication remains unknown. In this study, we examine genetic diversity and population structure in 215 plants from 11 countries in Mesoamerica, the Andes, and Amazonia using 25 nuclear microsatellite loci to propose an origin of domestication. Genetic analyses reveal one gene pool in Mesoamerica (Mexico) and four in South America (Brazilian Amazonia, Peruvian Amazonia and Andes, and Colombia), indicating greater differentiation among localities, possibly due to isolation between guava populations, particularly in the Amazonian and Andean regions. Moreover, Mesoamerican populations show high genetic diversity, with moderate genetic structure due to gene flow from northern South American populations. Dispersal scenarios suggest that Brazilian Amazonia is the probable origin of guava domestication, spreading from there to the Peruvian Andes, northern South America, Central America, and Mexico. These findings present the first evidence of guava domestication in the Americas, contributing to a deeper understanding of its evolutionary history.

## Introduction

In the Neotropics, there are approx. 8200 species either managed or domesticated to various degrees in Mesoamerica, the Andes, and the lowlands of South America, the majority of which are perennial^1^. This frequent use of long-lived species is due to their many valuable products such as roots, fleshy or starchy fruits, nuts, fibers, and oils^1–4^, which provide significant quantities of macro and micronutrients^2^. However, only a few of these perennial crops are known in international markets, and the majority are still cultivated mainly as subsistence crops for local consumption and sale^2,5^. Particularly in the Neotropics, fleshy fruits have been an essential dietary component of numerous human groups in pre- and post-Columbian times^6–8^. However, few studies on their genetic diversity and population structure have been performed, with some work on *Annona cherimola* Mill.^9^, *Bactris gasipaes* Kunth^10,11^, *Chrysophyllum cainito* L.^12,13^, *Carica papaya* L.^14^, *Spondias purpurea* L.^15^, and *Theobroma cacao* L.^16–18^. To bridge this knowledge gap and increase the number of Neotropical fruit species studied we conducted an analysis of the genetic diversity and population structure of guava.

Guava (*Psidium guajava* L.) is a Neotropical semi-domesticated fruit tree species of some importance in the Americas and elsewhere^19,20^. It is distributed from Mexico and the Antilles to northwestern Argentina^21^. The fruit is the most used part of the plant, consumed fresh or used to make candies, dried fruits, jams, jellies, juices, pastes, soup bases, and syrup^21,22^. It is a good source of calcium, iron, niacin, pantothenic acid, phosphorus, riboflavin, and thiamine^23^. In folk medicine, guava is used to treat respiratory discomfort, gastrointestinal problems and help to expel the placenta after childbirth^23,24^. Guava grows in tropical dry forests and savannah-like vegetation, as well as in disturbed areas (roadsides and grasslands), small agroecological environments (homegardens and orchards), and larger-scale production systems^21,22^. It adapts easily to different rainfall conditions and soil types but does not tolerate flooded soils and is sensitive to low temperatures^25^.

During post-Columbian times, guava was the fruit tree most widely recorded by European chroniclers of the sixteenth century, who documented its presence in Mesoamerica and South America in both reputably wild and cultivated populations^26^. The European conquerors learned to use guava fruits and leaves as medicine and food^8^, which prevail among indigenous peoples until now. The oldest archaeological record of macro remains place guava in pre-Columbian contexts in Southwestern Amazonia (dates between 9490 and 6505 calibrated years before present [cal. BP])^27^ and the human settlements of the Peruvian coast (7000 cal. BP)^28^. The earliest macro remains (fruit fragments) found in Mexico are much more recent, dating to ca. 670 cal. BP^29^.

Despite its cultural, economic, and historical importance, guava has received little attention from geneticists^30^. Perennial trees are often propagated asexually^3,4,31^, which results in a reduction of sexual reproduction^4,32^ and, therefore, slower rates of evolution and less pronounced changes in domestication syndrome traits^33,34^. Guava wild populations are very hard to distinguish from tolerated or feral individuals that may form small populations (personal observations). Without a clearly defined wild ancestor, it is difficult to identify centers of origin of domestication, quantify changes due to human selection and trace routes of human-mediated dispersal. Our study aims to characterize the genetic diversity and population structure of guava across parts of its Neotropical distribution using SSR markers, looking for answers to the following questions: (a) What is the level of genetic variation among the sampled genotypes of guava? (b) How is this diversity structured? (c) Is there isolation by distance between locations? (d) Where is the most likely origin of domestication? (e) What is the history of dispersal of this species?

## Results

### Null alleles, Hardy–Weinberg equilibrium, and linkage disequilibrium

We removed 18 samples and discarded the locus mPgCIR08 which had more than 40% of missing data, leaving 197 samples and 24 loci for further analysis. We found no evidence of null alleles in our data set. All loci showed significant deviations from Hardy–Weinberg equilibrium (HWE) and linkage disequilibrium (LD) in more than one population (Supplementary Tables 1, 2). Because the loci in HWE and LD were not the same for all localities, all markers were retained for further analyses. The genotype accumulation curve (Supplementary Fig. 1) shows that the set of loci tested had sufficient power to discriminate between individuals. The curve revealed that 100% of the genotypes could be detected with 12 markers, hence the loci accurately estimated the diversity of our sample.

### Genetic diversity and genetic differentiation

**Figure 1:**
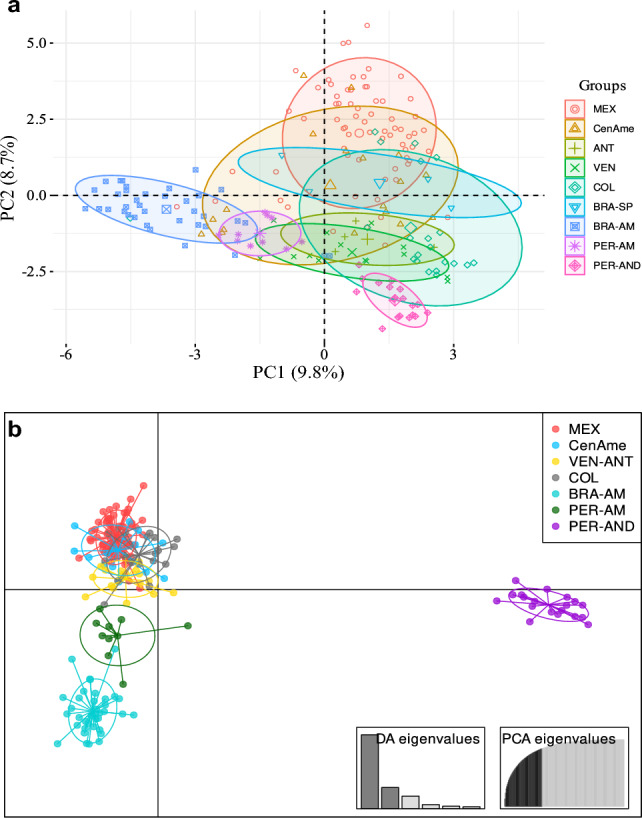
(**a**) Principal component analysis (PCA) of microsatellite genotype data from *P. guajava* individuals showing the clustering along principal component axis 1–2. (**b**) Discriminant analysis of principal components (DAPC) for eight guava localities. Localities: MEX (Mexico), CenAme (Central America), ANT (The Antilles), VEN (Venezuela), COL (Colombia), BRA-SP (São Paulo, Brazil), BRA-AM (Brazilian Amazonia), PER-AM (Peruvian Amazonia), PER-AND (Peruvian Andes).

The PCA provided evidence of genetic structure of guava across its geographical range. The first two principal components explained 18.5% of the total variation (Fig. 1a). Amazonian guavas from Brazil and Peru (BRA-AM and PER-AM, respectively) formed well-defined clusters. In contrast, guava samples from Colombia (COL) and Venezuela (VEN) overlap with those from the Antilles (ANT), documenting the close relationship between these regions. Given that the centroids of the Colombian and Venezuelan clusters do not co-occur within their respective standard deviation ellipses, we decided to define the Colombian (COL) and Venezuela-Antilles (VEN-ANT) clusters separately. Similarly, we defined a Peruvian Andes (PER-AND) cluster, Central American (CenAme) cluster and a Mexican (MEX) cluster. We decided to discard the samples from southern Brazil (BRA-SP), because the origin of these samples is uncertain (Fig. 1a). Therefore, for subsequent analyses, we used 192 samples.

The DAPC analysis with 7 defined groups from the results of the PCA revealed a clear differentiation of guavas from Brazilian and Peruvian Amazonia and the Peruvian Andes (Fig. 1b). These results were consistent with the patterns obtained using PCA but with far clearer differentiations among clusters. Likewise, the Mesoamerican (MEX + CenAme) and the northern South American clusters (COL + VEN-ANT) appeared as admixed groups (Fig. 1b). We performed a second DAPC analysis excluding the Peruvian and Brazilian well-differentiated groups to depict the relationships among Mesoamerican and northern South American groups. This analysis showed that VEN-ANT cluster was well differentiated from COL, CenAme and MEX, which are more closely related (Supplementary Fig. 2).

The Mexican and Central American localities showed the highest *H*_*E*_ values, while the lowest *H*_*E*_ values were found in the Peruvian Andes and Peruvian Amazonia (Table 1). When considering all the samples, guava showed high values of total and unbiased expected heterozygosity, and low values of average observed heterozygosity (*H*_*O*_) (Table 1). The results for each locality maintain this pattern. The inbreeding coefficient showed high values for most of the localities (Table 1).

**Table 1:** Genetic diversity obtained with 24 nuclear microsatellite loci for 192 plants of *P. guajava* from seven localities*.*

	N	A	A_r_	P_Ar_	*H*_*O*_	*H*_*E*_	u*H*_*E*_	F
Species	192	351	14.25	5.14	0.30 ± 0.015	0.78 ± 0.020	0.79 ± 0.021	0.46 ± 0.025
DAPC								
MEX	69	227	1.70	0.51	0.34 ± 0.024	0.70 ± 0.022	0.70 ± 0.022	0.52 ± 0.028
CenAme	18	152	1.71	0.44	0.34 ± 0.027	0.70 ± 0.026	0.71 ± 0.027	0.50 ± 0.043
VEN-ANT	18	151	1.67	0.44	0.26 ± 0.026	0.65 ± 0.046	0.68 ± 0.047	0.50 ± 0.052
COL	21	142	1.60	0.35	0.21 ± 0.023	0.60 ± 0.041	0.61 ± 0.042	0.61 ± 0.044
BRA-AMA	37	158	1.61	0.59	0.25 ± 0.026	0.60 ± 0.046	0.61 ± 0.046	0.53 ± 0.044
PER-AMA	10	83	1.50	0.62	0.44 ± 0.062	0.47 ± 0.047	0.53 ± 0.055	0.06 ± 0.093
PER-AND	19	81	1.45	0.56	0.22 ± 0.042	0.44 ± 0.048	0.45 ± 0.050	0.50 ± 0.071

*N* Number of individuals, *A* number of alleles, *Ar* rarefied allelic richness, *P*_*Ar*_ number of private alleles, *H*_*O*_ observed heterozygosity, *H*_*E*_ expected heterozygosity, u*H*_*E*_ unbiased heterozygosity, *F* fixation index.

**Figure 2:**
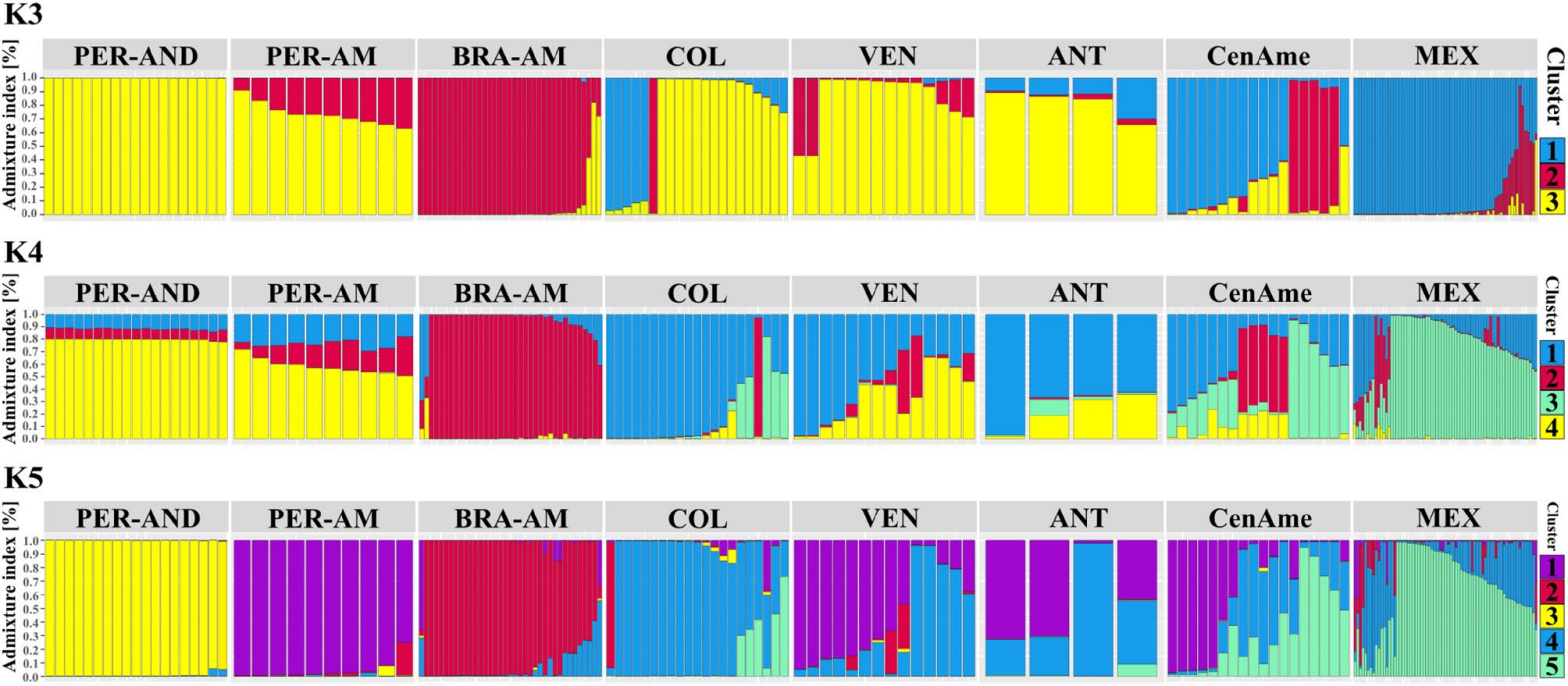
Assignment probabilities of each of the 192 guava samples to each cluster inferred by STRUCTURE for K = 3, 4, and 5. Each sample is represented by a vertical bar, and color indicates the probability of belonging to each cluster. Samples are ordered according to the geographic region from southern to northern parts of the Americas.

The results of the STRUCTURE analysis were consistent with the results from PCA and DAPC. Evanno and Jane's methods indicated an optimal value of K = 3 and K = 5 as the most likely numbers of genetic clusters (Supplementary Fig. 3). In K = 3, three genetic clusters are probable. Cluster 1 is predominant in Mexico, strongly represented in Central America, moderately represented in Colombia, and a minor component of Venezuela and the Antilles (Fig. 2). Cluster is 2 predominant in Brazilian Amazonia and a relatively minor component of all other localities. The Peruvian Andes, the only locality with 100% of the plants fully attributed to cluster 3 (Fig. 2), was strongly represented in Peruvian Amazonia, Colombia, Venezuela, and the Antilles, and present in Brazilian Amazonia, Central America, and Mexico. At K = 4, although it is not an optimal value according to Evanno and Jane's methods, a new genetic cluster appears in Colombia, foreshadowing K = 5 (Fig. 2). In K = 5, each of the five clusters is dominant in a separate locality: (1) Peruvian Amazonia; (2) Brazilian Amazonia; (3) Peruvian Andes; (4) Colombia; (5) Mexico. Venezuela and the Antilles are mixtures of Peruvian Amazonia and Colombia, while Central America is a mixture of Peruvian Amazonia, Colombia, and Mexico (Fig. 2).

This sequence of clusters from K = 3 to K = 5 suggests that western South America (BRA-AM → PER-AM → PER-AND) contains the origin of domesticated guava, given the number of clusters dominated by distinct genetic groups. From western South America, guava was then dispersed northward through Colombia to Central America and Mexico. Colombia is also the crossroads for the guava that arrived in Venezuela and later the Antilles. The fact that Mexico is always a clear cluster suggests that dispersal happened long enough ago for the thorough mixing of origins that became a distinct genetic group.

Estimates of Wright's F among the sampling localities indicated that guava diversity is higher within localities than among localities; nevertheless, we found intermediate levels of genetic differentiation with F_*ST*_ = 0.21 (Supplementary Table 3). *F*_*IT*_ (0.64) and *F*_*IS*_ (0.54) estimates were higher compared to *F*_*ST*_ (Supplementary Table 3). All fixation indexes were statistically significant. These results suggest that the frequency of heterozygotes is lower than expected under HWE.

**Figure 3:**
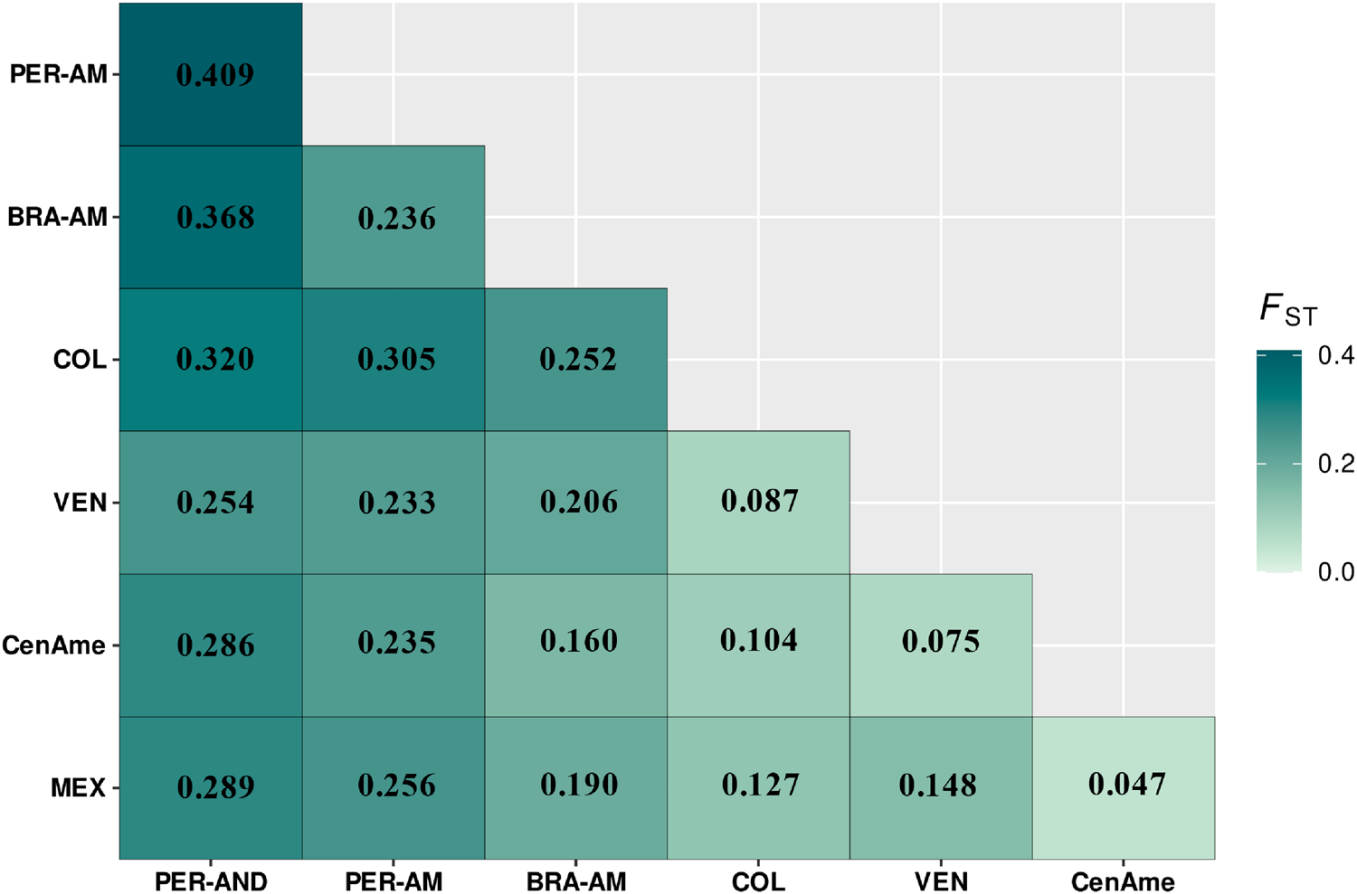
*F*_ST_ genetic differentiation values among the 192 samples of guava grouped by localities.

The pairwise F_*ST*_ values suggested moderate to high genetic differentiation between localities (Fig. 3). The largest difference was observed for the Peruvian Andes, Peruvian Amazonia, and Brazilian Amazonia. Mesoamerica (MEX and CenAme) and northern South America (COL and VEN-ANT) showed less pairwise genetic differentiation (Fig. 3).

The Mantel test revealed no significant correlation between genetic and geographic distance matrices (R^2^ = − 0.287, p = 0.779), indicating a lack of isolation by distance. We found that PER-AND, PER-AM, and BRA-AM localities, which are geographically closer to each other, are genetically less similar (Supplementary Fig. 4; Supplementary Table 4). The observed low pairwise *F*_*ST*_ values indicates possible long-distance gene flow between MEX, CenAme, VEN, and COL (Supplementary Tables 4–5). According to AMOVA, the variation among samples within localities (41%, *Φ* = 0.52) is higher than between localities (21.04%, *Φ* = 0.62; Table 2).

**Table 2:** Results of the analysis of molecular variance (AMOVA) testing for differentiation among localities in *P. guajava.*

	*df*	Sum of squares	% variance	*Φ*-statistic	*p*-value
Among localities	6	1235.802	21.04	0.627	0.001
Among samples within localities	1811	3884.253	41.64	0.527	0.001
Within samples	188	1248.343	37.32	0.210	0.001
Total	375	6368.399	100		

**Figure 4:**
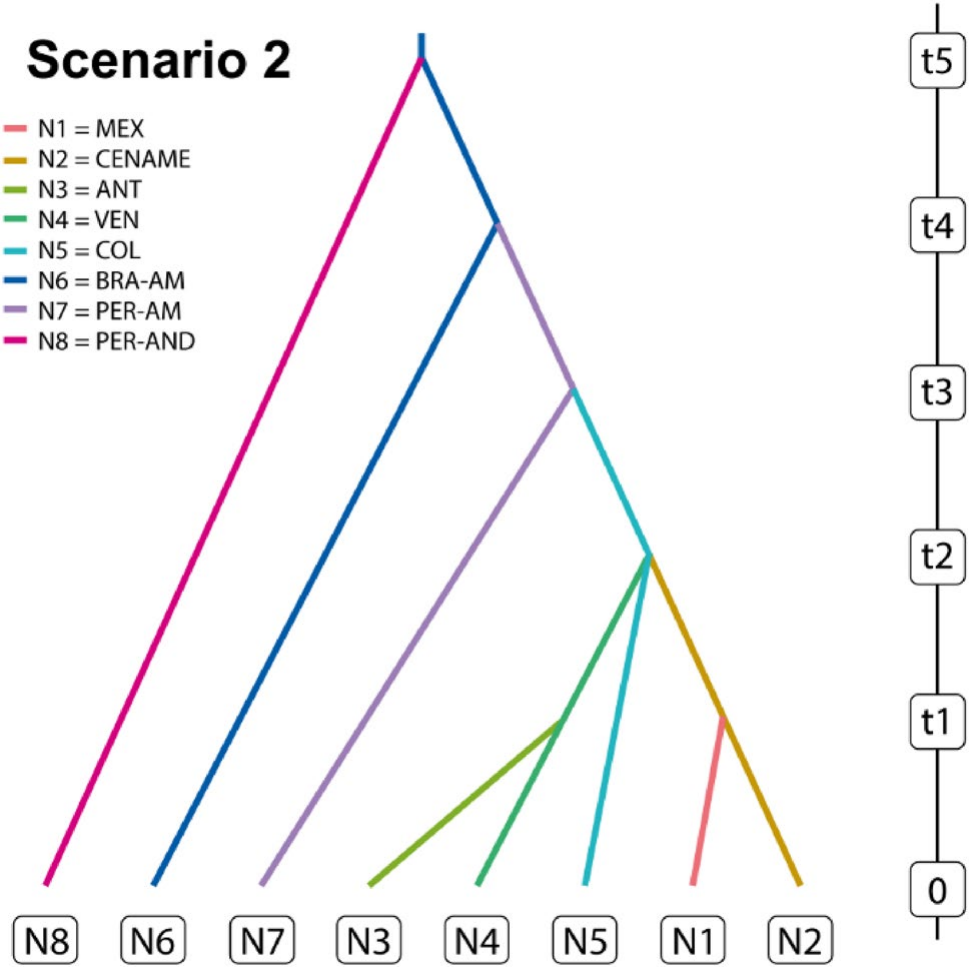
Highest-probability scenario tested for dispersal of *Psidium guajava* in the Neotropics. Eight localities with effective population sizes N1 to N8 correspond to MEX (Mexico), CenAme (Central America), ANT (The Antilles), VEN (Venezuela), COL (Colombia), BRA-AM (Brazilian Amazonia), PER-AM (Peruvian Amazonia), PER-AND (Peruvian Andes), respectively. Time since divergence corresponds to t1 to t5.

Approximate Bayesian Computation (ABC) analyses indicated that the best supported dispersal hypothesis was scenario 2 (Fig. 4) with posterior probability of 0.999 and non-overlapping confidence intervals. This scenario showed low type I and type II error rates (0.00013; Supplementary Table 6), suggesting that domestication started in South America, specifically in Brazilian Amazonia (Brazil-AM), with dissemination to Mexico via Peruvian Amazonia (PER-AMA) and northern South America (COL).

## Discussion

Perennial trees, characterized by their extended lifespan and delayed sexual reproduction, tend to exhibit a weak population structure^35,36^. Domestication profoundly influences the population dynamics and genetic structure of species, shaped by a complex set of evolutionary events involving both natural factors and ancestral and contemporary human activities. The present study provides the first evidence of the domestication history of guava in the Neotropics and assesses scenarios of the species' dispersal in the region.

Our analyses found that values of guava genetic diversity expressed as *H*_*E*_ ranged from 0.44 to 0.70. These levels are comparable to those of other perennials with populations domesticated to some degree, such as *A. cherimola*, *Olea europaea* L., and *Prunus armeniaca* L.^9,37,38^. However, compared to other *Psidium* species, *P. guajava* has high genetic diversity, for example, in a single population in Southeast Brazil the maximum *H*_*E*_ values (*H*_*E*_ = 0.71) are comparable to those of *P. guineense* Sw. (*H*_*E*_ = 0.74) and *P. macahense* O. Berg (*H*_*E*_ = 0.63)^39^. In contrast, an insular *Psidium* species (*P. galapageium* Hook. f.) has moderate to low (*H*_*E*_ = 0.275–0.570) genetic diversity^40^. *P. cattleianum* Afzel. ex Sabine also showed lower diversity values (*H*_*E*_ = 0.117–0.326)^41^.

Observed heterozygosity (*Ho*) systematically showed lower values than expected heterozygosity, suggesting heterozygote deficiency among guava due to inbreeding. In different islands of the Galapagos^42^ and guava samples from a germplasm bank^43^ registered similar results. These findings may be explained, in part, by self-fertilization and vegetative propagation, which can occur in *P. guajava*^44^. Likewise, Robertson’s^45^ hypotheses could be useful to explain the heterozygosity decline in guava. He proposes that subdividing a population into several isolated groups would allow maximum genetic diversity (minimum global co-ancestry) to be achieved in the long term since different allelic variants will develop and become fixed in each group, becoming a genetic reservoir of variation. However, complete isolation leads to higher rates of local inbreeding with the possible consequence of inbreeding depression. Therefore, Robertson^45^ also suggests that occasional mixing of these subpopulations would minimize the overall rate of inbreeding. In support of this hypothesis, we found lower rates of global inbreeding for guava. Likewise, Mantel's analysis suggests long-distance gene flow, especially between the populations of northern South America (COL and VEN) and Mesoamerica (MEX and CenAme; Supplementary Tables 4–5), which would allow the reduction of inbreeding and its effects. Overall, the absence of isolation by distance, the broad range of F_*ST*_ values, the high F_*IS*_ value, and the separate gene pools indicated by PCA, DAPC, and STRUCTURE suggest a metapopulation dynamic. Local cultivated guava populations may originate from the surrounding genetic variation and occasionally receive long-distance gene flow. Finally, further studies are needed to examine the cause of heterozygote deficiency in guava.

Contrary to our hypothesis of south-to-north decrease in diversity, the genetic diversity pattern, expressed as *H*_*E*_ is just the opposite. A decreasing trend in *H*_*E*_ was observed from Mexico and Central America (*H*_*E*_ = 0.70) to the Peruvian Andes (PER-AND = 0.44). This pattern can be explained by the mixture of guavas from different regions occurring in Central America and Mexico. In these areas, most of the individuals show signatures of admixture of well-defined South American genetic groups. Hence, anthropic dispersal may have enhanced guava genetic diversity in Central America and Mexico. In addition, the diversity of environmental conditions, new biotic interactions, and selection pressures in Mesoamerica could have contributed to the maintenance of genetic variants that were present in the gene pool due to mutations. These events would explain an increased guava genetic diversity in response to new environmental conditions and challenges, a hypothesis that is testable by using ecological niche models^46–48^. In addition, whether this pattern points towards a center of genetic diversity or is the result of admixture among clusters is a matter to be evaluated by rating explicit demographic scenarios.

In our study, the genetic differentiation of *P. guajava* populations yielded an *F*_*ST*_ value of 0.207, indicating moderate differentiation, considering the wide geographical range across which the species is distributed. Likewise, the molecular variance is higher among individuals/within localities than among localities. Similar findings have been reported for other perennial fruit trees like *A. cherimola*, *Diospyros kaki* L.F., *Juglans regia* L., *Mangifera indica* L., *O. europaea*, *P. persica* L., and *P*. *armeniaca*^9,37,38,49–52^. In the case of guava, the observed *F*_*ST*_ value may be attributed to limited gene flow among the sampled localities, which span different regions of the Neotropics. Similarly, the pattern of variance identified here can be due to outcrossing and guava’s invasive (successional) character (Hamrick et al.^53^ and therein). In cultivated and invasive populations of guava, the genetic variation pattern is also similar, with higher genetic variance among individuals/between populations and clearly defined genetic groups^42,54,55^.

Regarding the genetic clustering found in our study, each of the most geographically isolated populations from South America (Peruvian Andes [PER-AND], and Brazilian and Peruvian Amazonia [BRA-AM; PER-AMA]) belongs to a distinct genetic group and shows greater differentiation in relation to other groups (*F*_*ST*_; Fig. 3). Localities in northern South America, Central America, and Mesoamerica show lower values of genetic differentiation with some individuals being admixed. This scenario suggests a pattern of greater differentiation among localities in South America, probably due to the isolation between guava populations, especially between the Amazonian and Andean regions. In Amazonia, the vast expanse of tropical rainforest and relatively homogeneous climatic conditions have favored the domestication of a variety of crops such as *Manihot esculenta* Crantz, *T. cacao*, and various fruits and nuts. This region is been an independent center of plant domestication, where indigenous peoples have managed and cultivated numerous crops over millennia, resulting in notable genetic diversity within these crops^1,6,27,56,57^. In contrast, the Andean ecosystems' altitudinal and climatic variability has led to the genetic differentiation of plants adapted to specific microenvironments. This environmental diversity has promoted the evolution of plants with unique genetic traits necessary for surviving extreme conditions, resulting in a mosaic of locally adapted crops, each with distinct genetic variations^1,6,27,56,57^. Moreover, human interaction with the environment in both regions has played a crucial role. In Amazonia, landscape management practices, such as the creation of "terra preta" (Amazonian Dark Earths), have enriched the soil and fostered crop diversification. In the Andes, agricultural techniques such as terracing and irrigation, have enabled the adaptation and cultivation of plants on steep slopes and less fertile soils^1,57,58^.

Therefore, the localities evaluated here would have likely been exposed to specific evolutionary processes, considering the climatic and ecological characteristics of their geographical origin’s setting, thus promoting differentiation between them. Variable admixture levels among populations may also be the outcome of diverse trade routes and human migrations over time^30^, as is the case of *J. regia*^50^ and *M. indica*^51^.

According to the best-supported ABC scenario, Amazonia is the most probable area of domestication of guava, and the first dispersal route likely was from there towards the Peruvian Andes. This result agrees with the oldest archaeological guava macro remains found in Southwestern Amazonia in the Teotonio archeological site, in a layer between 9490 and 6505 cal. BP^27^. In South America, the lowlands of southwestern Amazonia are recognized as a relevant center of domestication^1,27,56,57^ and the place from which important species, such as manioc and peanut (*Arachis hypogaea* L.), dispersed towards the Peruvian dry coast^58^. Indeed, a significant number of archaeological guava remains dating from 6975 to 450 BP have been reported from the Peruvian dry coast (see Fig. 4 in Arévalo-Marín et al.^30^). This evidence also supports the hypothesis that guava could have spread through the Andes, from Amazonia to the Peruvian coast, as the best supported scenario in this study suggests. Therefore, more detailed archaeological and genetic studies that include samples from both the southwestern region and other areas of Amazonia would allow for a confirmation of the domestication area of guava.

In summary, our study provides an overview of the genetic diversity and population structure of guava in the Neotropics. The microsatellite markers and Bayesian clustering approaches identified the presence of one gene pool in Mesoamerican (Mexico) and four in South America (Brazilian Amazonia, Peruvian Amazonia and Andes, and Colombia). The high genetic differentiation between the Brazilian and Peruvian Amazonia and Peruvian Andes guava samples could be due to environmental differences, since guava subpopulations in distinct geographic settings may reflect divergent local adaptation. Niche analyses are needed to understand whether climatic events could explain these hypotheses, and genomic analyses would allow testing of hypotheses of local adaptation. On the other hand, the ABC approach identified Brazilian Amazonia as the potential area of guava domestication, with subsequent dispersal into western and northern South America and Mesoamerica where local diversification processes occurring in these last two regions could also underlie the observed diversity patterns. Follow-up studies that include defined populations of feral and cultivated guavas, and focused sampling in southwestern Amazonia and the Andes could help to unravel the guava domestication process.

## Materials and methods

### Material

We studied 215 guava plants from 11 countries. We collected 86 samples from Brazil, Colombia, Honduras, Mexico, and Venezuela in the guava germplasm bank of the Instituto Nacional de Investigaciones Forestales, Agrícolas y Pecuarias (INIFAP) in Aguascalientes, Mexico; 17 samples from the guava collection of the Tropical Agricultural Research and Higher Education Center (CATIE) in Turrialba, Costa Rica, from Costa Rica, El Salvador, Guatemala, and Honduras; and 26 samples from Brazil, Colombia, and Puerto Rico in the guava collection of the Corporación Colombiana de Investigación Agropecuaria (Agrosavia) in Palmira, Colombia. We also included samples collected outside germplasm banks from Brazilian Amazonia (38 plants), Peruvian Amazonia (15 plants), the Peruvian Andes (19 plants), and 14 samples from different localities in Venezuela. We considered samples collected in the Peruvian Andes and Brazilian Amazonia as tolerated or planted because these were collected in empty lots, roadsides, orchards, and gardens. Samples from Venezuela and Peruvian Amazonia were collected in areas far from plantations or crops; however, since it is difficult to distinguish between wild and feral guavas, each of these samples were considered feral. Because of this collection strategy, which is commonly used with cultivated plants, we are not dealing with biological populations, so we will call groups of plants from different areas “localities”. All methods were performed in accordance with the relevant guidelines and regulations, and appropriate permissions for the collection of plant material were obtained from all relevant parties.

### Molecular methods

DNA was extracted from young leaves using a CTAB-based protocol^59^. Initially, all 215 individuals were genotyped using 25 nuclear microsatellite loci developed for *P. guajava*^60,61^. We combined five primers in each of the five multiplex reactions (see Supplementary Table 7 for primers and multiplex reaction details). PCRs were performed using the Platinum Multiplex PCR Master Mix (Thermo-Fisher, USA) following the manufacturer’s instructions for reaction assembly and program. Every reaction was driven to a 5.5 μL final volume containing 2.0 μL Platinum Multiplex PCR Master Mix, 2.0 μL PCR grade H_2_O, 0.5 μL G/C enhancer volume, 1.0 μL DNA template (50–200 ng/μL), and primer concentrations between 50 to 70 nM according to each product’s relative fluorescent units (RFU). Multiplex reactions required an annealing temperature of 55 °C for all primers; 40 cycles were used in every PCR reaction. When amplification was not successful, we repeated the PCR reactions using 0.04 μL Kapa polymerase (Kapa Taq HotStart), 2.0 μL Buffer Kapa, 2.0 μL PCR grade H_2_O, and 1.0 μL DNA template (50–200 ng/μL). The annealing temperature and the number of cycles were maintained. To prevent possible contamination, we used negative controls for each multiplex assembly. All products were verified in 2% agarose gel electrophoresis. PCRs were carried out in a MultiGene OptiMax (Labnet International, Inc., Edison, NJ, USA) or in a 2700 thermal cycler (Applied Biosystems, Foster City, CA, USA). Genotyping was achieved using the Microsatellite plugin (v. 1.4.7) of Geneious Prime 2022 (Dotmatics, NZ). Allele scoring was performed manually following Selkoe and Toonen^62^.

### Null alleles, Hardy–Weinberg equilibrium, and linkage disequilibrium tests

We tested the presence and frequency of null alleles following Brookfield^63^ using the *PopGenReport* v.3.0.7 package^64^ in R. We calculated deviations from Hardy–Weinberg equilibrium (HWE) for each locus and separately for each locality. Also, we calculated HWE across all samples using the ‘hw.test’ function of the R package *pegas* v.1.1^65^, with 1000 Monte Carlo permutations. Alpha levels to determine statistically significant deviations from Hardy–Weinberg proportions and independent sorting of genotypes were adjusted using the false discovery rate (FDR) approach developed by Benjamini and Hochberg^66^, using 0.05 alpha level. P-values were corrected for multiple comparisons using the Benjamini–Hochberg method^66^. We calculated a measure of correlation (*r̄*_*d*_^67^ using the function ‘ia’^68^ in the R package *poppr* v.2.9.3, for testing overall linkage disequilibrium. Using the function ‘genotype curve’ of the same package, we described the genotypic diversity in relation to different combinations of loci by a genotype accumulation curve to determine whether our sample provided a reasonable estimate of genetic diversity. The curve was generated by randomly sampling x loci and counting the number of multilocus genotypes (MLG) observed. This sampling was repeated r times from 1 to n − 1 loci, creating n − 1 distributions of observed MLGs^69^.

### Genetic diversity and genetic differentiation

Genetic differentiation was examined using several complementary approaches. First, as an exploratory method, we performed a Principal Components Analysis (PCA) to summarize the genetic variation based on the microsatellite data set. Subsequently, we performed a Discriminant Analysis of Principal Components (DAPC)^70^. DAPC is an approach that optimizes the separation of individuals into predefined groups using a discriminant function of the principal component^70^. Based on DAPC, the membership probability was calculated for the overall genetic background of an individual. We used the components identified in the PCA analysis as predefined groups for the DAPC implementation. For implementing the PCA, we used the ‘dudi.pca’ function from *ade4* v.1.7–22 R^71^ and visualized it with *factoextra* v.1.0.7 R^72^. For DAPC, we used *adegenet* v.2.2.10^73^ implemented in R.

We assessed standard measures of genetic diversity for the entire dataset and genetic groups according to DAPC results. The number of individuals (*N*), number of alleles (*A*), and the expected (*H*_E_) and observed (*H*_O_) heterozygosities were calculated using *poppr* v.2.9.3^68^ in R. We estimated rarefied allelic richness using the ‘allel.rich’ function of *PopGenReport* v.3.0.7^64^ in R. Private allele richness (*A*P) were calculated using a rarefaction approach^74,75^ implemented in ADZE 1.0^76^.

As an additional test to calculate the group assignment probability for each sample, we performed genetic population structure analysis using the Bayesian approach implemented in STRUCTURE 2.3.4^77,78^, based on the admixture model with correlated allele frequencies and information on the origin of localities (popinfo = 1). The admixture model was tested for K-values ranging from 1 to 8, since 8 is the number of sampled regions, with 10 independent runs per K value for the entire dataset. We used 1,000,000 Markov Chain Monte Carlo iterations with a burn-in length of 100,000. To determine the most probable value of K, we used Evanno’s ΔK method^79^ and mean LnP(K)^80^ implemented in Structure Harvester v0.6.94^81^. We used CLUMPP 1.1.2^82^ with the Greedy algorithm to infer the optimal K-cluster affiliations of samples and StructuRly v.0.1.0^83^ in R to generate bar graphs of the STRUCTURE software results.

Wright's F statistics^84^ (*F*_*IS*_, *F*_*IT*_, and *F*_*ST*_) were estimated using the methods of Weir and Cockerham^85^. We also calculated the genetic differentiation among localities through a pairwise *F*_*ST*_ matrix. Both the F statistics and the paired* F*_*ST*_ matrix were calculated with 95% confidence intervals from 10,000 bootstrapping, using the ‘diffCalc’ function of *diveRsity*^86^. A Mantel test^87^ was used to assess isolation by distance (IBD) between pairs of guava localities. We used the geographic distance matrix transform from coordinates in Euclidean distance and calculated using the function ‘dist’ in the *stats* v.4.3.1 in R and a linearized pairwise *F*_*ST*_ matrix (*F*_*ST*_/1 − *F*_*ST*_) as genetic distance. The function ‘mantel.rtest’ from *ade4* v.1.7-22^88^ was used to calculate the Mantel test, and scatter-plots were then generated with *adegenet* v.2.2.10^73^.

We also tested the degree of genetic differentiation between DAPC groups (determined here) and locations, performing the analysis of molecular variance (AMOVA) followed by an estimation of the extent of genetic differentiation with phi-statistics, both using the ‘poppr.amova’ function in *poppr* v.2.9.3^68^. The significance of variance components was assessed using a permutation test implemented through the ‘randtest’ function in *ape4* v.5.7^71,89,90^ with 999 permutations.

### Identification of the origin of domestication

We used nuclear microsatellite data to run the Approximate Bayesian Computation (ABC) framework^91,92^ implemented in DIYABC-RF GUI^93^ to model a possible branching order among guava localities that would represent the history of domestication of the lineage. We considered five scenarios (1) Mexico as a probable center of origin of domestication with dissemination to South America; (2) South America, specifically Brazilian Amazonia (Brazil-AM) as a probable center and later dissemination to Mexico via Peru; (3) Two independent centers of origin of domestication, one in the Peruvian Andes (Peru-An) and another in Mexico; (4) Peruvian Amazonia (Peru-AM) and Brazil-AM as independents centers of origin of domestication and dissemination towards northern South America with Central America and Mexico being of admixed destination; and (5) domestication in northern South America and dissemination to three areas (Mexico and Central America, Venezuela and Antilles, and Peru and Brazil) (Supplementary Fig. 5). The priors and conditions for each parameter can be found in the Supplementary Material 2; we considered a generation time of 10 years (probable fruiting time in natural conditions)^44^. We conducted previous runs to adjust the tested scenarios and the parameters^94^. For the final run, we obtained 500,000 simulated datasets, 500 trees, and 424 summary statistics. To identify the best supported scenario, we performed model check based on 500 pseudo-observed data sets (PODs) under each scenario to assess confidence in scenario choice, and to estimate the class specific error rates, which is the mean classification error rate^93,95,96^.

### Supplementary Information

Supplementary Information.

## Data Availability

The genotyping data generated in the present study will be released upon acceptance and is (privately) available at: https://figshare.com/s/47366c57067686695d91.
