# Supplementary material for: Genetic analyses and dispersal patterns unveil the Amazonian origin of guava domestication
Source: Sci Rep. 2024 Jul 8;14:15755. doi: 10.1038/s41598-024-66495-y (PMC11231237; doi:10.1038/s41598-024-66495-y)
Supplement: Supplementary file 1 — Supplementary Information. [file 41598_2024_66495_MOESM1_ESM.pdf]

SUPPLEMENTARY MATERIAL 1

Genetic analyses and dispersal patterns unveil the Amazonian origin of guava domestication

E. Arévalo-Marín, A. Casas, H. Alvarado-Sizzo, E. Ruiz-Sanchez, G. Castellanos-Morales, L. Jardón-Barbolla, G. Fermin, J. S. Padilla-Ramírez, C.R. Clement

Supplementary Figures:

Supplementary Figure 1. Genotype accumulation curve for 197 samples of *P. guajava* over 24 loci.

Supplementary Figure 2. Discriminant analysis of principal components (DAPC) for only 4 guava localities.

Supplementary Figure 3. Graphical method for the detection of K's groups

Supplementary Figure 4. Mantel test of isolation by distance (IBD). The relationship is not significant.

Supplementary Figure 5. Evolutionary scenarios under comparison using ABC analyses.

Supplementary Tables:

Supplementary Table 1. *p-values* of the Hardy–Weinberg equilibrium test with the FRD-based correction for each combination of sample and locus for country.

Supplementary Table 2. The proportions of loci that are out of HWE for each population and standardized index of association (LD).

Supplementary Table 3. Estimates of Wright's F-statistics obtained for 192 samples of *P. guajava* using 24 specific microsatellite loci.

Supplementary Table 4. Geographic distances *Psidium guajava* populations.

Supplementary Table 5. Genetic distances (F_{ST}) *Psidium guajava* populations.

Supplementary Table 6. Class specific prediction errors for 5 scenarios tested with DIYABC-RF.

Supplementary Table 7. Characteristics of the 25 microsatellite loci used in the analysis of genetic diversity of guava (*Psidium guajava*) in the Americas

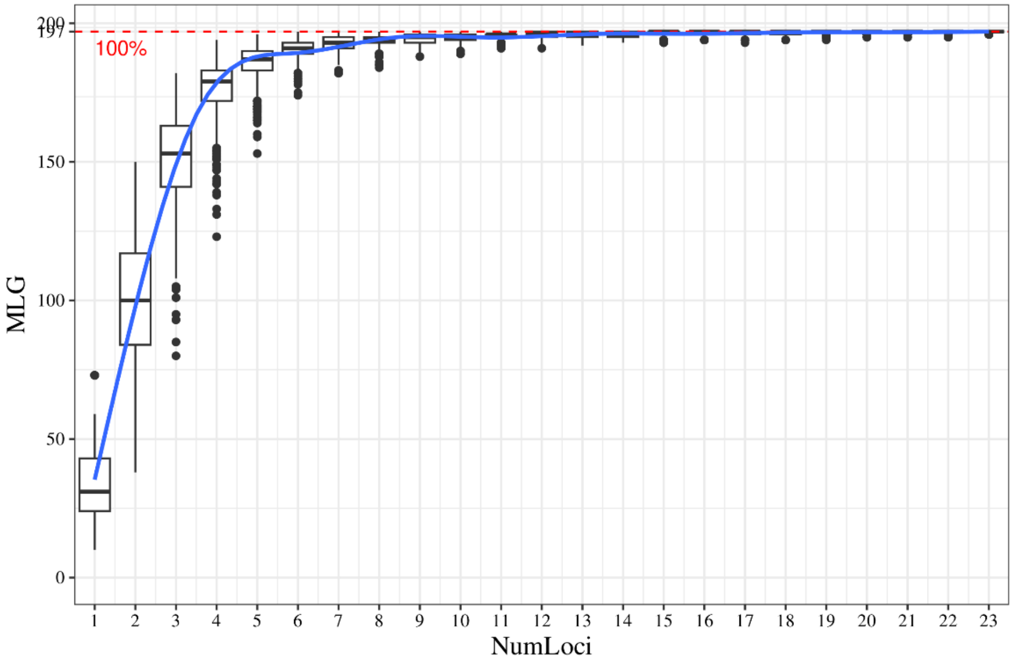

Supplementary Fig. 1. Genotype accumulation curve for 197 samples of *P. guajava* over 24 loci. The horizontal axis represents the number of loci randomly sampled without replacement up to n-1 loci, and the vertical axis shows the number of unique multilocus genotypes observed in the data set. The level of 100% of the unique multilocus genotype detected is indicated with a dotted red line.

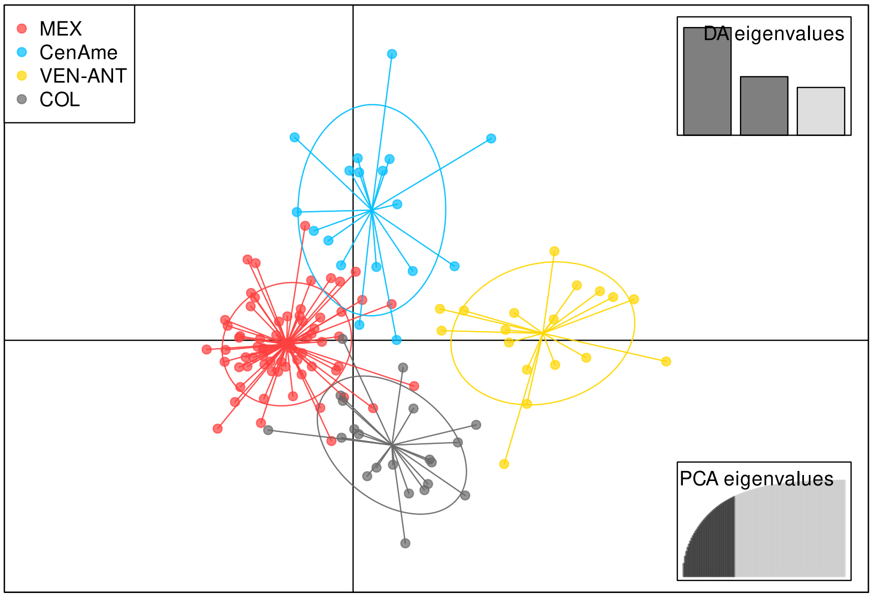

Supplementary Fig. 2. Discriminant analysis of principal components (DAPC) for only 4 guava localities. Localities: MEX (Mexico), CenAme (Central America), ANT (The Antilles), VEN (Venezuela), COL (Colombia), BRA-SP (São Paulo, Brazil), BRA-AM (Brazilian Amazonia), PER-AM (Peruvian Amazonia), PER-AND (Peruvian Andes).

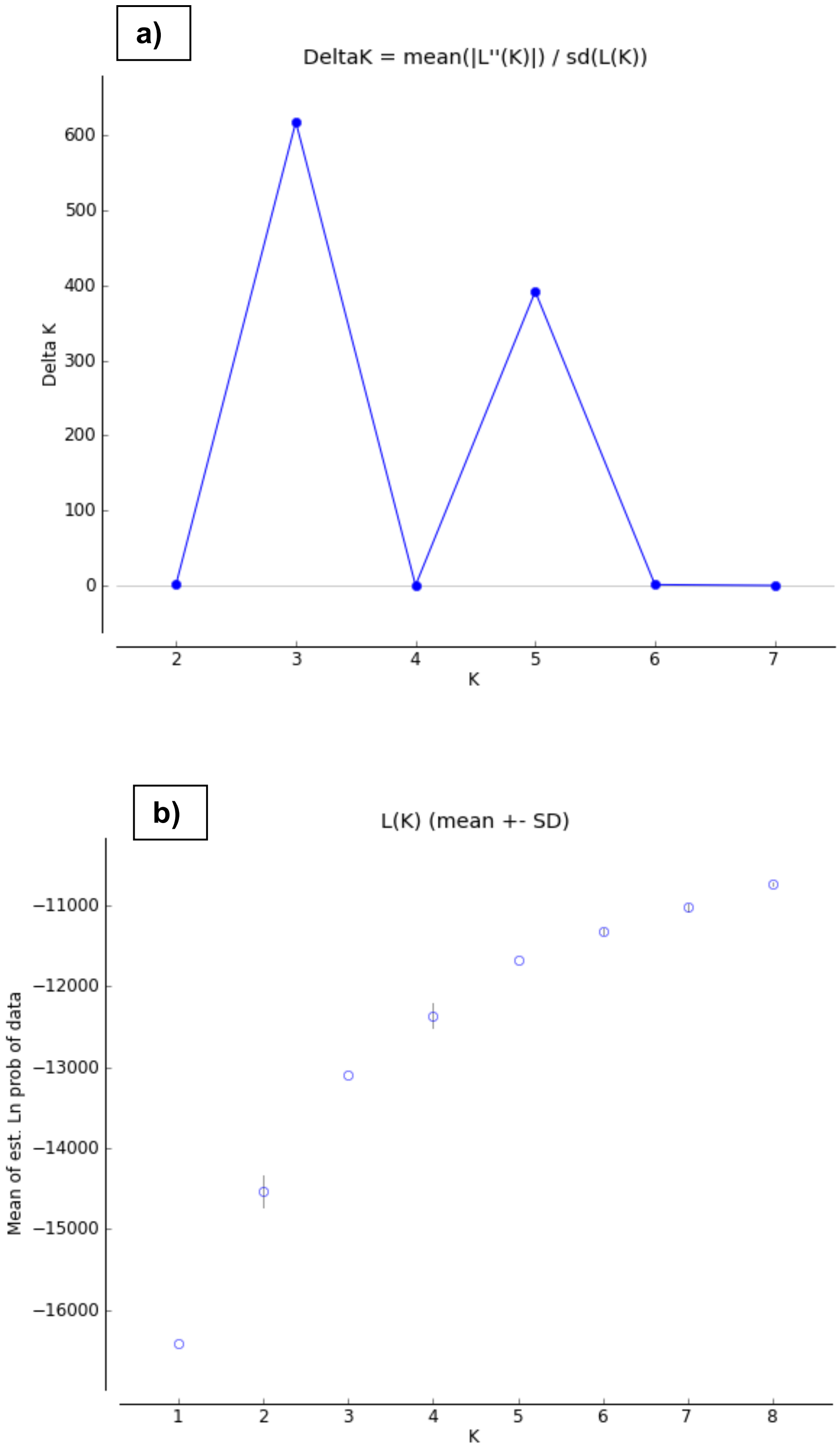

Supplementary Fig. 3. Graphical method allowing the detection of the number of groups K using a) ΔK, and b) LnP(K).

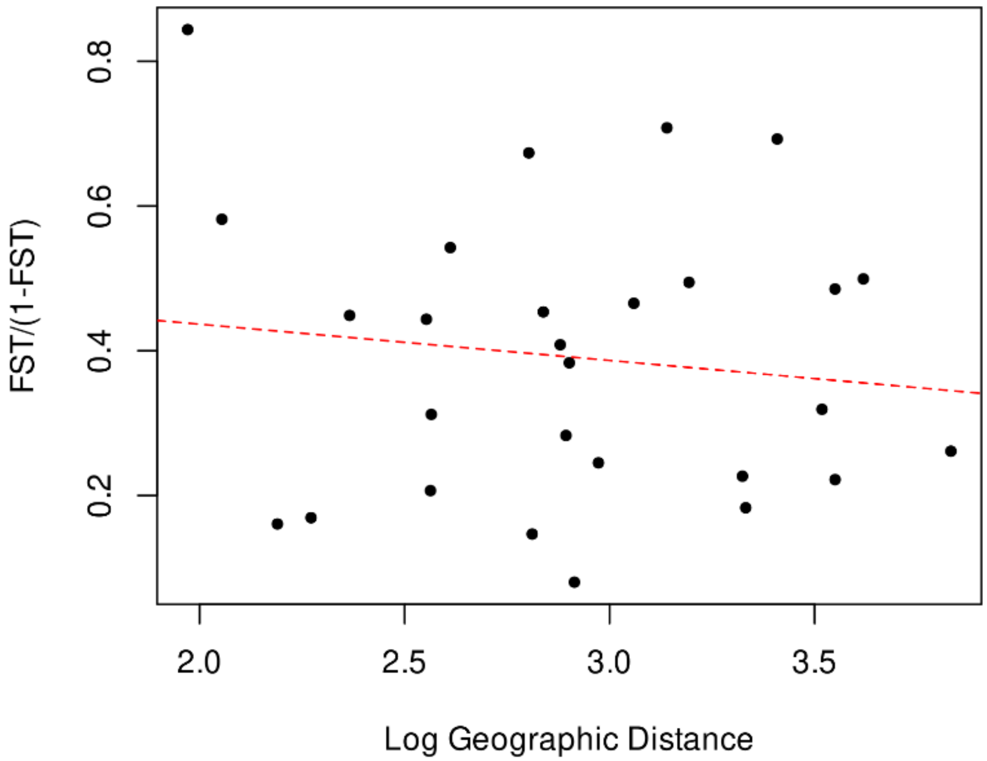

Supplementary Fig. 4. Mantel test of isolation by distance (IBD). The relationship is not significant.

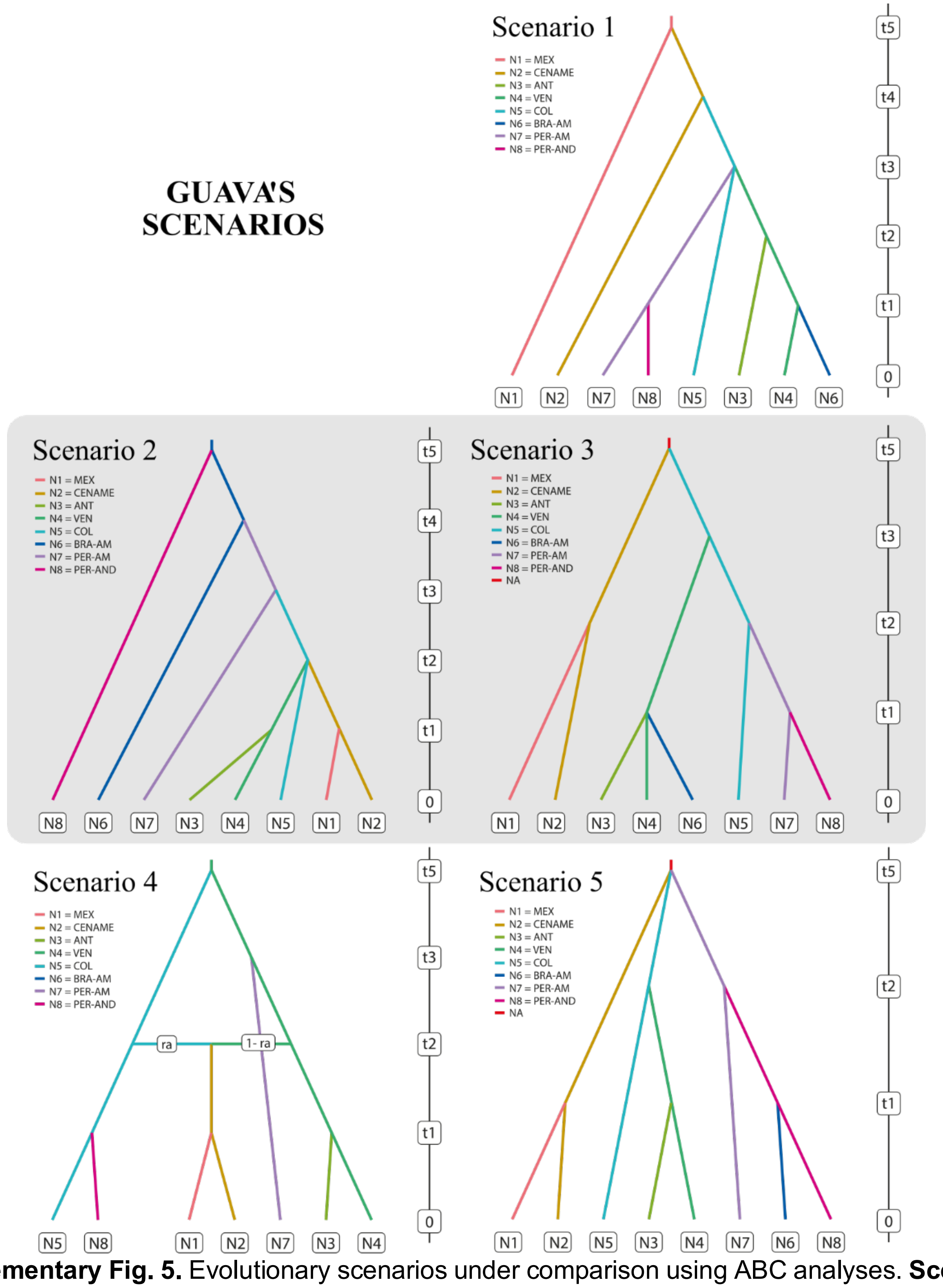

Supplementary Fig. 5. Evolutionary scenarios under comparison using ABC analyses. **Scenario 1**: Mexico as a probable domestication; **scenario 2:** South America (Brazil-AM) as a probable area of domestication; **scenario 3:** Two independent events of domestication (Peru-An and Mexico); **scenario 4:** Peru-AM and Brazil-AM as independents areas of domestication; and **scenario 5**: domestication in northern South America.

Supplementary Table 1. *p-values* of the Hardy–Weinberg equilibrium test with the FRD-based correction for each combination of sample and locus for country

Chisq	MEX	CenAme	ANT	VEN	COL	BRA-SP	BRA-AM	PER-AM	PER-AND
mPgCIR137	**0.00**	**0.01**	0.31	**0.00**	**0.00**	0.17	**0.00**	**0.09**	**0.04**
mPgCIR25	**0.00**	0.29	0.68	**0.00**	**0.00**	0.31	**0.00**	0.25	**0.00**
mPgCIR7	**0.00**	**0.00**	0.35	**0.00**	**0.00**	1.00	**0.00**	**0.00**	**0.00**
mPgCIR420	**0.00**	**0.00**	0.35	**0.06**	**0.00**	0.10	**0.00**	**0.03**	**0.00**
mPgCIR11	**0.00**	**0.00**	0.24	**0.00**	**0.00**	0.14	**0.00**	**0.02**	0.94
mPgCIR 437	**0.00**	**0.00**	0.70	**0.04**	**0.00**	0.25	0.70	0.73	**0.00**
mPgCIR 9	**0.00**	**0.00**	0.17	0.28	**0.00**	**0.06**	**0.00**	0.57	**0.00**
mPgCIR14	**0.00**	0.58	1.00	0.88	1.00	**0.01**	**0.00**	0.84	1.00
mPgCIR1	**0.00**	**0.00**	0.11	0.24	0.22	0.78	**0.00**	0.32	0.41
mPgCIR209	**0.00**	**0.00**	**0.05**	**0.03**	**0.00**	**0.06**	**0.00**	1.00	0.07
mPgCIR15	**0.00**	**0.00**	0.08	**0.00**	**0.00**	**0.05**	**0.00**	0.93	**0.00**
mPgCIR2	**0.00**	**0.00**	0.78	0.89	**0.00**	0.41	0.93	0.87	**0.00**
mPgCIR22	**0.00**	**0.04**	0.16	**0.02**	**0.00**	**0.02**	**0.00**	0.14	**0.00**
mPgCIR20	**0.00**	**0.01**	0.56	**0.00**	0.38	**0.05**	**0.00**	**0.00**	0.39
mPgCIR237	**0.00**	**0.06**	**0.05**	**0.06**	**0.00**	0.11	**0.00**	0.58	**0.00**
mPgCIR4	**0.00**	**0.00**	0.16	0.42	**0.00**	0.70	0.67	0.81	0.24
mPgCIR26	**0.00**	0.20	0.17	**0.00**	**0.00**	1.00	**0.00**	0.91	**0.01**
mPgCIR17	**0.00**	**0.00**	0.16	0.12	**0.00**	**0.02**	**0.00**	0.17	**0.01**
mPgCIR10	**0.00**	**0.00**	**0.08**	**0.00**	**0.00**	0.14	**0.00**	0.80	**0.01**
mPgCIR101	**0.00**	**0.00**	1.00	**0.00**	**0.00**	**0.08**	**0.00**	0.16	0.91
mPgCIR21	**0.00**	**0.00**	0.70	**0.01**	**0.00**	**0.01**	**0.00**	0.33	1.00

mPgCIR243	**0.00**	**0.00**	0.70	**0.00**	**0.00**	**0.04**	**0.00**	0.11	**0.00**
mPgCIR19	**0.00**	**0.03**	0.24	**0.00**	**0.02**	**0.05**	**0.00**	0.88	**0.00**
mPgCIR13	**0.00**	**0.00**	0.73	0.99	**0.00**	**0.02**	**0.00**	0.72	**0.00**

MC	MEX	CenAme	ANT	VEN	COL	BRA-SP	BRA-AM	PER-AM	PER-AND
mPgCIR137	**0.00**	**0.00**	0.32	**0.00**	**0.00**	**0.02**	**0.00**	0.23	**0.03**
mPgCIR25	**0.00**	0.10	1.00	**0.00**	**0.00**	0.22	**0.00**	1.00	**0.00**
mPgCIR7	**0.00**	**0.00**	0.43	**0.00**	**0.00**	1.00	**0.00**	**0.01**	**0.01**
mPgCIR420	**0.00**	**0.00**	0.48	**0.01**	**0.00**	**0.09**	**0.00**	**0.00**	**0.00**
mPgCIR11	**0.00**	**0.01**	0.21	**0.00**	**0.00**	**0.05**	**0.00**	**0.01**	0.72
mPgCIR 437	**0.00**	**0.00**	1.00	**0.00**	**0.00**	0.16	0.60	1.00	**0.00**
mPgCIR 9	**0.00**	**0.00**	0.32	**0.06**	**0.01**	**0.09**	**0.00**	1.00	**0.00**
mPgCIR14	**0.00**	0.48	1.00	1.00	1.00	0.10	**0.00**	1.00	1.00
mPgCIR1	**0.00**	**0.00**	0.17	**0.03**	0.10	1.00	**0.00**	1.00	0.14
mPgCIR209	**0.00**	**0.00**	0.16	**0.00**	**0.00**	**0.01**	**0.00**	1.00	**0.02**
mPgCIR15	**0.00**	**0.00**	0.18	**0.00**	**0.00**	**0.00**	**0.00**	1.00	**0.00**
mPgCIR2	**0.00**	**0.00**	1.00	1.00	**0.00**	1.00	1.00	1.00	**0.00**
mPgCIR22	**0.00**	**0.00**	0.35	**0.00**	**0.00**	**0.02**	**0.00**	0.12	**0.00**
mPgCIR20	**0.00**	**0.00**	1.00	**0.00**	**0.01**	0.14	**0.03**	**0.06**	0.19
mPgCIR237	**0.00**	**0.00**	0.14	**0.01**	**0.00**	0.10	**0.00**	1.00	**0.00**
mPgCIR4	**0.00**	**0.00**	0.31	0.37	**0.00**	1.00	**0.40**	0.86	0.33
mPgCIR26	**0.00**	0.18	**0.07**	**0.00**	**0.00**	1.00	**0.00**	1.00	**0.02**
mPgCIR17	**0.00**	**0.00**	0.32	**0.00**	**0.00**	**0.02**	**0.00**	0.13	**0.00**
mPgCIR10	**0.00**	**0.00**	0.20	**0.00**	**0.00**	**0.05**	**0.00**	0.59	**0.02**
mPgCIR101	**0.00**	**0.00**	1.00	**0.00**	**0.00**	**0.03**	**0.00**	0.16	1.00

mPgCIR21	**0.00**	**0.00**	1.00	**0.00**	**0.00**	**0.00**	**0.00**	0.39	1.00
mPgCIR243	**0.00**	**0.00**	1.00	**0.00**	**0.00**	**0.04**	**0.00**	**0.03**	**0.00**
mPgCIR19	**0.00**	**0.00**	0.18	**0.00**	**0.04**	**0.00**	**0.00**	0.96	**0.00**
mPgCIR13	**0.00**	**0.00**	1.00	1.00	**0.00**	**0.01**	**0.00**	0.54	**0.00**

Chisq: p-values calculated using the exact χ2-test, MC: p-values calculated using the Monte Carlo permutation test. p-values ≤ 0.05 are in bold, and cells with p-values <0.05 for both HWE tests are boxed.

Supplementary Table 2. The proportions of loci that are out of HWE for each population and standardized index of association (LD)

	Hardy-Weinberg equilibrium				Linkage equilibrium	
	**Chisq**	**MC**	**Chisq.fdr**	**MC.fdr**	**rbarD**	**p.rD**
**DAPC clusters**						
MEX	1.00	1.00	1.00	1.00	0.08	0.001
CenAme	0.83	0.88	0.75	0.88	0.19	0.001
ANT	0.08	0.00	0.00	0.00	0.11	0.026
VEN	0.63	0.79	0.54	0.79	0.06	0.02
COL	0.88	0.92	0.88	0.88	0.10	0.001
BRA-SP	0.38	0.50	0.21	0.38	0.40	0.001
BRA-AMA	0.88	0.88	0.88	0.88	0.06	0.023
PER-AMA	0.17	0.17	0.17	0.17	0.03	0.199
PER-AND	0.67	0.71	0.63	0.67	0.05	0.002

Chisq: values calculated using the exact χ2- test; *MC*: values calculated using the Monte Carlo permutation test; *.fdr indicates testing with Benjamini-Hochberg correction; significance of rbarD ($p < 0.01$)

Supplementary Table 3. Estimates of Wright's F-statistics obtained for 192 samples of *P. guajava* using 24 specific microsatellite loci.

**DAPC Groups**	F_{IS}	F_{ST}	F_{IT}
All loci	0.5433	0.2079	0.6383
Upper (CI95%)	0.5844	0.2319	0.6702
Lower (CI95%)	0.5013	0.1859	0.6055

CI95% = 95% confidence interval.

Supplementary Table 4. Geographic distances *Psidium guajava* populations

	MEX	CenAme	ANT	VEN	COL	BRA-AM	PER-AM
CenAme	18.433242						
ANT	33.717281	19.545013					
VEN	34.810855	16.629832	12.975978				
COL	27.995193	9.698104	18.057741	8.933477			
BRA-AM	46.173604	27.769857	24.376937	13.002596	18.205155		
PER-AM	34.805722	17.086784	23.098807	10.653623	7.801922	12.847361	
PER-AND	37.295852	21.309452	30.230732	17.806803	13.618934	16.49345	7.177258

Supplementary Table 5. Genetic distances (F_{ST}) *Psidium guajava* populations

	MEX	CenAme	ANT	VEN	COL	BRA-AM	PER-AM
CenAme	2.914156						
ANT	3.51801	2.97272					
VEN	3.549929	2.811198	2.5631				
COL	3.332033	2.27193	2.893574	2.189806			
BRA-AM	3.832408	3.323951	3.193637	2.565149	2.901705		
PER-AM	3.549782	2.838305	3.139781	2.3659	2.05437	2.553138	
PER-AND	3.618882	3.059151	3.408859	2.879581	2.611461	2.802963	1.970917

Supplementary Table 6. Class specific prediction errors for 5 scenarios tested with DIYABC-RF. Model performance indicated by type I (rows) and type II (columns) errors in percentage of times each scenario was predicted.

	Scenario 1	Scenario 2	Scenario 3	Scenario 4	Scenario 5	Class error
Predicted 1	99.96	0.00	0.04	0.00	0.00	0.00035
Predicted 2	0.00	99.99	0.00	0.00	0.00	7.97×10^{-05}
Predicted 3	0.00	0.00	100.00	0.00	0.00	9.97×10^{-06}
Predicted 4	0.00	0.01	0.00	99.98	0.01	0.00021
Predicted 5	0.00	0.00	0.00	0.00	100.00	0

Supplementary Table 7. Characteristics of the 25 microsatellite loci used in the analysis of genetic diversity of guava (*Psidium guajava*) in the Americas

SSR Locus	Repeat Motif	Primer sequences (5' to 3')		Fragment size range
		Forward	Reverse	
mPgCIR1 **(B)**	$(GA)_{17}$	TAGTGCTTTGGTTGCTT	GCAGGTGGATATAAGGTC	236–250
mPgCIR2 **(C)**	$(GA)_{20}$	AGTGAACGACTGAAGACC	ATTACACATTCAGCCACTT	202–230
mPgCIR4 **(D)**	$(GA)_{25}$	TTCAGGGTCTATGGCTAC	CAACAAGATACAGCGAACT	126–150
mPgCIR7 **(A)**	$(CA)_{13}AA(GAA)_3$	ATGGAGGTAGGTTGATG	CGTAGTAATCGAAGAAATG	148–160
mPgCIR8 **(B)***	$(GA)_{12}$	ACTTTCGGTCTCAACAAG	AGGCTTCCTACAAAAGTG	210-224
mPgCIR 9 **(B)**	$(GA)_{19}$	GCGTGTCGTATTGTTTC	ATTTTCTTCTGCCTTGTC	156–176
mPgCIR10 **(D)**	(CT)12	GTTGGCTCTTATTTTGGT	GCCCCATATCTAGGAAG	262–320
mPgCIR11 **(A)**	(CT)17	TGAAAGACAACAAACGAG	TTACACCCACCTAAATAAGA	298–314
mPgCIR13 **(E)**	$AC)_{12}(AT)_4G(GA)_2$	CCTTTTTCCCGACCATTACA	TCGCACTGAGATTTTGTGCT	240–260
mPgCIR14 **(B)**	$(GA)_{11}$	TAAACACAACAAGGGTCA	CAGTTTTCATATCGTCCTC	184–186
mPgCIR15 **(C)**	$(GA)_8GG(GA)_9$	TCTAATCCCCTGAGTTTC	CCGATCATCTCTTTCTTT	144–172
mPgCIR17 **(D)**	$(CT)_{23}$	CCTTTCGTCATATTCACTT	CATTGGATGGTTGACAT	230–240
mPgCIR19 **(E)**	$(CT)_{16}$	AAAATCCTGAAGACGAAC	TATCAGAGGCTTGCATTA	258–280
mPgCIR20 **(C)**	$(CT)_{14}(CA)_{17}$	TATACCACACGCTGAAAC	TTCCCCATAAACATCTCT	270–298
mPgCIR21 **(E)**	$(AG)_{15}GG(AG)_7$	TGCCCTTCTAAGTATAACAG	AGCTACAAACCTTCCTAAA	150–164
mPgCIR22 **(C)**	$(GT)_9(GA)_{14}$	CATAAGGACATTTGAGGAA	AATAAGAAAGCGAGCAGA	236–252
mPgCIR25 **(A)**	$(GA)_{24}$	GACAATCCAATCTCACTTT	TGTGTCAAGCATACCTTC	104–130
mPgCIR26 **(D)**	$(GT)_2(GA)_{17}$	CTACCAAGGAGATAGCAAG	GAAATGGAGACTTTGGAG	180–198

mPgCIR101 **(E)**	$(GT)_{10}$	ATGGCTGTAAGAAGCAAAAG	GAAGAAATGTAGGTGCGTTC	110
mPgCIR137 **(A)**	$(GA)_{18}$	GGGGAATGCAGAGATTGT	AGATGATGGTCTCGCTTTT	100
mPgCIR209 **(C)**	$(GA)_{15}$	CTAAAGCCACATCCAGCA	CTAACATTTGCCTTCTACAGC	139
mPgCIR237 **(D)**	$(GA)_{16}$	AGATTCCATCTGCGATTGT	GCGGATCAAAACCTAATCT	101
mPgCIR243 **(E)**	$(GA)_{29}$	ACAGCAGGACACAAAGGA	GCTCTGAGGTGGTTTTCAT	174
mPgCIR420 **(A)**	$(CA)_{15}$	CAACTTTGCTAGAGATGAAGC	ATGTAGTAATCGAAGAAATGGTT	239
mPgCIR 437 **(B)**	$(AC)_{10}$	ACAACAGTTCTGATCCCAAA	CTCGGAGACACAGAGGTCTA	153

*SRR locus not used in final analyzes (see results)

SUPPLEMENTARY MATERIAL 2

Genetic analyses and dispersal patterns unveil the Amazonian origin of guava domestication

E. Arévalo-Marín, A. Casas, H. Alvarado-Sizzo, E. Ruiz-Sanchez, G. Castellanos-Morales, L. Jardón-Barbolla, G. Fermin, J. S. Padilla-Ramírez, C.R. Clement

Supplementary Material 2. The priors and conditions for each parameter in the ABC analysis of *Psidium guava.*

Supplementary Material 2.The priors and conditions for each parameter in the ABC analysis of Psidium guava.

```
micros24_guava_Mod .mss
15 parameters and 424 summary statistics

5 scenarios: 16 16 17 16 17
scenario 1 [0.2] (13)
N1 N2 N3 N4 N5 N6 N7 N8
0 sample 1
0 sample 2
0 sample 3
0 sample 4
0 sample 5
0 sample 6
0 sample 7
0 sample 8
t1 merge 7 8
t1 merge 4 6
t2 merge 4 3
t3 merge 5 4
t3 merge 5 7
t4 merge 2 5
t5 merge 1 2
scenario 2 [0.2] (13)
N1 N2 N3 N4 N5 N6 N7 N8
0 sample 1
0 sample 2
0 sample 3
0 sample 4
0 sample 5
0 sample 6
0 sample 7
0 sample 8
t1 merge 2 1
t1 merge 4 3
t2 merge 5 2
t2 merge 5 4
t3 merge 7 5
t4 merge 6 7
t5 merge 6 8
scenario 3 [0.2] (13)
N1 N2 N3 N4 N5 N6 N7 N8
0 sample 1
0 sample 2
0 sample 3
0 sample 4
0 sample 5
0 sample 6
0 sample 7
```

```
0 sample 8
t1 merge 4 6
t1 merge 4 3
t1 merge 7 8
t2 merge 5 7
t2 merge 2 1
t3 merge 5 4
t5 merge 2 5
t5 varNe 2 Na
scenario 4 [0.2] (13)
N1 N2 N3 N4 N5 N6 N7 N8
0 sample 1
0 sample 2
0 sample 3
0 sample 4
0 sample 5
0 sample 6
0 sample 7
0 sample 8
t1 merge 4 3
t1 merge 2 1
t1 merge 5 8
t2 split 2 5 4 ra
t3 merge 4 7
t3 merge 4 6
t5 merge 4 5
scenario 5 [0.2] (12)
N1 N2 N3 N4 N5 N6 N7 N8
0 sample 1
0 sample 2
0 sample 3
0 sample 4
0 sample 5
0 sample 6
0 sample 7
0 sample 8
t1 merge 2 1
t1 merge 4 3
t1 merge 8 6
t2 merge 5 4
t2 merge 7 8
t5 merge 7 5
t5 merge 7 2
t5 varNe 7 Na

historical parameters priors (15,7)
N1 N UN[10,60000,0,0]
N2 N UN[10,10000,0,0]
N3 N UN[10,5000,0,0]
N4 N UN[10,15000,0,0]
```

```
N5 N UN[10,10000,0,0]
N6 N UN[10,10000,0,0]
N7 N UN[10,5000,0,0]
N8 N UN[10,5000,0,0]
t1 T UN[10,1500,0,0]
t2 T UN[10,5000,0,0]
t3 T UN[10,15000,0,0]
t4 T UN[10,100000,0,0]
t5 T UN[10,100000,0,0]
Na N UN[10,1000000,0,0]
ra A UN[0.001,0.999,0,0]
t5>t2
t2>=t1
t5>=t3
t3>=t2
t5>=t4
t4>=t3
t3>=t1
DRAW UNTIL

loci description (24)
mPgCIR137 <A> [M] G1 2 40
mPgCIR25 <A> [M] G1 2 40
mPgCIR7 <A> [M] G1 2 40
mPgCIR420 <A> [M] G1 2 40
mPgCIR11 <A> [M] G1 2 40
mPgCIR437 <A> [M] G1 2 40
mPgCIR9 <A> [M] G1 2 40
mPgCIR14 <A> [M] G1 2 40
mPgCIR1 <A> [M] G1 2 40
mPgCIR209 <A> [M] G1 2 40
mPgCIR15 <A> [M] G1 2 40
mPgCIR2 <A> [M] G1 2 40
mPgCIR22 <A> [M] G1 2 40
mPgCIR20 <A> [M] G1 2 40
mPgCIR237 <A> [M] G1 2 40
mPgCIR4 <A> [M] G1 2 40
mPgCIR26 <A> [M] G1 2 40
mPgCIR17 <A> [M] G1 2 40
mPgCIR10 <A> [M] G1 2 40
mPgCIR101 <A> [M] G1 2 40
mPgCIR21 <A> [M] G1 2 40
mPgCIR243 <A> [M] G1 2 40
mPgCIR19 <A> [M] G1 2 40
mPgCIR13 <A> [M] G1 2 40

group priors (1)
group G1 [M]
MEANMU UN[1.00E-004,1.00E-3,0.0005,2]
GAMMU GA[1.00E-005,1.00E-002,Mean_u,2]
```

```
MEANP UN[1.00E-001,3.00E-001,0.22,2]
GAMP GA[1.00E-002,9.00E-001,Mean_P,2]
MEANSNI LU[1.00E-008,1.00E-005,1.00E-007,2]
GAMSNI GA[1.00E-009,1.00E-004,Mean_u_SNI,2]

group summary statistics (424)
group G1 (424)
NAL 1 2 3 4 5 6 7 8
HET 1 2 3 4 5 6 7 8
VAR 1 2 3 4 5 6 7 8
MGW 1 2 3 4 5 6 7 8
N2P 1.2 1.3 1.4 1.5 1.6 1.7 1.8 2.3 2.4 2.5 2.6 2.7 2.8 3.4 3.5 3.6
3.7 3.8 4.5 4.6 4.7 4.8 5.6 5.7 5.8 6.7 6.8 7.8
H2P 1.2 1.3 1.4 1.5 1.6 1.7 1.8 2.3 2.4 2.5 2.6 2.7 2.8 3.4 3.5 3.6
3.7 3.8 4.5 4.6 4.7 4.8 5.6 5.7 5.8 6.7 6.8 7.8
V2P 1.2 1.3 1.4 1.5 1.6 1.7 1.8 2.3 2.4 2.5 2.6 2.7 2.8 3.4 3.5 3.6
3.7 3.8 4.5 4.6 4.7 4.8 5.6 5.7 5.8 6.7 6.8 7.8
FST 1.2 1.3 1.4 1.5 1.6 1.7 1.8 2.3 2.4 2.5 2.6 2.7 2.8 3.4 3.5 3.6
3.7 3.8 4.5 4.6 4.7 4.8 5.6 5.7 5.8 6.7 6.8 7.8
LIK 1.2 2.1 1.3 3.1 1.4 4.1 1.5 5.1 1.6 6.1 1.7 7.1 1.8 8.1 2.3 3.2
2.4 4.2 2.5 5.2 2.6 6.2 2.7 7.2 2.8 8.2 3.4 4.3 3.5 5.3 3.6 6.3 3.7
7.3 3.8 8.3 4.5 5.4 4.6 6.4 4.7 7.4 4.8 8.4 5.6 6.5 5.7 7.5 5.8 8.5
6.7 7.6 6.8 8.6 7.8 8.7
DAS 1.2 1.3 1.4 1.5 1.6 1.7 1.8 2.3 2.4 2.5 2.6 2.7 2.8 3.4 3.5 3.6
3.7 3.8 4.5 4.6 4.7 4.8 5.6 5.7 5.8 6.7 6.8 7.8
DM2 1.2 1.3 1.4 1.5 1.6 1.7 1.8 2.3 2.4 2.5 2.6 2.7 2.8 3.4 3.5 3.6
3.7 3.8 4.5 4.6 4.7 4.8 5.6 5.7 5.8 6.7 6.8 7.8
AML 1.2.3 2.1.3 3.1.2 1.2.4 2.1.4 4.1.2 1.2.5 2.1.5 5.1.2 1.2.6 2.1.6
6.1.2 1.2.7 2.1.7 7.1.2 1.2.8 2.1.8 8.1.2 1.3.4 3.1.4 4.1.3 1.3.5
3.1.5 5.1.3 1.3.6 3.1.6 6.1.3 1.3.7 3.1.7 7.1.3 1.3.8 3.1.8 8.1.3
1.4.5 4.1.5 5.1.4 1.4.6 4.1.6 6.1.4 1.4.7 4.1.7 7.1.4 1.4.8 4.1.8
8.1.4 1.5.6 5.1.6 6.1.5 1.5.7 5.1.7 7.1.5 1.5.8 5.1.8 8.1.5 1.6.7
6.1.7 7.1.6 1.6.8 6.1.8 8.1.6 1.7.8 7.1.8 8.1.7 2.3.4 3.2.4 4.2.3
2.3.5 3.2.5 5.2.3 2.3.6 3.2.6 6.2.3 2.3.7 3.2.7 7.2.3 2.3.8 3.2.8
8.2.3 2.4.5 4.2.5 5.2.4 2.4.6 4.2.6 6.2.4 2.4.7 4.2.7 7.2.4 2.4.8
4.2.8 8.2.4 2.5.6 5.2.6 6.2.5 2.5.7 5.2.7 7.2.5 2.5.8 5.2.8 8.2.5
2.6.7 6.2.7 7.2.6 2.6.8 6.2.8 8.2.6 2.7.8 7.2.8 8.2.7 3.4.5 4.3.5
5.3.4 3.4.6 4.3.6 6.3.4 3.4.7 4.3.7 7.3.4 3.4.8 4.3.8 8.3.4 3.5.6
5.3.6 6.3.5 3.5.7 5.3.7 7.3.5 3.5.8 5.3.8 8.3.5 3.6.7 6.3.7 7.3.6
3.6.8 6.3.8 8.3.6 3.7.8 7.3.8 8.3.7 4.5.6 5.4.6 6.4.5 4.5.7 5.4.7
7.4.5 4.5.8 5.4.8 8.4.5 4.6.7 6.4.7 7.4.6 4.6.8 6.4.8 8.4.6 4.7.8
7.4.8 8.4.7 5.6.7 6.5.7 7.5.6 5.6.8 6.5.8 8.5.6 5.7.8 7.5.8 8.5.7
6.7.8 7.6.8 8.6.7

scenario N1 N2 N3 N4 N5 N6 N7 N8 t1 t2 t3 t4 t5 Na ra µmic_1 pmic_1
snimic_1   NAL_1_1       NAL_1_2       NAL_1_3       NAL_1_4
NAL_1_5       NAL_1_6       NAL_1_7       NAL_1_8       HET_1_1
HET_1_2       HET_1_3       HET_1_4       HET_1_5       HET_1_6
HET_1_7       HET_1_8       VAR_1_1       VAR_1_2       VAR_1_3
VAR_1_4       VAR_1_5       VAR_1_6       VAR_1_7       VAR_1_8
```

MGW_1_1	MGW_1_2	MGW_1_3	MGW_1_4	MGW_1_5
MGW_1_6	MGW_1_7	MGW_1_8	N2P_1_1.2	N2P_1_1.3
N2P_1_1.4	N2P_1_1.5	N2P_1_1.6	N2P_1_1.7	N2P_1_1.8
N2P_1_2.3	N2P_1_2.4	N2P_1_2.5	N2P_1_2.6	N2P_1_2.7
N2P_1_2.8	N2P_1_3.4	N2P_1_3.5	N2P_1_3.6	N2P_1_3.7
N2P_1_3.8	N2P_1_4.5	N2P_1_4.6	N2P_1_4.7	N2P_1_4.8
N2P_1_5.6	N2P_1_5.7	N2P_1_5.8	N2P_1_6.7	N2P_1_6.8
N2P_1_7.8	H2P_1_1.2	H2P_1_1.3	H2P_1_1.4	H2P_1_1.5
H2P_1_1.6	H2P_1_1.7	H2P_1_1.8	H2P_1_2.3	H2P_1_2.4
H2P_1_2.5	H2P_1_2.6	H2P_1_2.7	H2P_1_2.8	H2P_1_3.4
H2P_1_3.5	H2P_1_3.6	H2P_1_3.7	H2P_1_3.8	H2P_1_4.5
H2P_1_4.6	H2P_1_4.7	H2P_1_4.8	H2P_1_5.6	H2P_1_5.7
H2P_1_5.8	H2P_1_6.7	H2P_1_6.8	H2P_1_7.8	V2P_1_1.2
V2P_1_1.3	V2P_1_1.4	V2P_1_1.5	V2P_1_1.6	V2P_1_1.7
V2P_1_1.8	V2P_1_2.3	V2P_1_2.4	V2P_1_2.5	V2P_1_2.6
V2P_1_2.7	V2P_1_2.8	V2P_1_3.4	V2P_1_3.5	V2P_1_3.6
V2P_1_3.7	V2P_1_3.8	V2P_1_4.5	V2P_1_4.6	V2P_1_4.7
V2P_1_4.8	V2P_1_5.6	V2P_1_5.7	V2P_1_5.8	V2P_1_6.7
V2P_1_6.8	V2P_1_7.8	FST_1_1.2	FST_1_1.3	FST_1_1.4
FST_1_1.5	FST_1_1.6	FST_1_1.7	FST_1_1.8	FST_1_2.3
FST_1_2.4	FST_1_2.5	FST_1_2.6	FST_1_2.7	FST_1_2.8
FST_1_3.4	FST_1_3.5	FST_1_3.6	FST_1_3.7	FST_1_3.8
FST_1_4.5	FST_1_4.6	FST_1_4.7	FST_1_4.8	FST_1_5.6
FST_1_5.7	FST_1_5.8	FST_1_6.7	FST_1_6.8	FST_1_7.8
LIK_1_1.2	LIK_1_2.1	LIK_1_1.3	LIK_1_3.1	LIK_1_1.4
LIK_1_4.1	LIK_1_1.5	LIK_1_5.1	LIK_1_1.6	LIK_1_6.1
LIK_1_1.7	LIK_1_7.1	LIK_1_1.8	LIK_1_8.1	LIK_1_2.3
LIK_1_3.2	LIK_1_2.4	LIK_1_4.2	LIK_1_2.5	LIK_1_5.2
LIK_1_2.6	LIK_1_6.2	LIK_1_2.7	LIK_1_7.2	LIK_1_2.8
LIK_1_8.2	LIK_1_3.4	LIK_1_4.3	LIK_1_3.5	LIK_1_5.3
LIK_1_3.6	LIK_1_6.3	LIK_1_3.7	LIK_1_7.3	LIK_1_3.8
LIK_1_8.3	LIK_1_4.5	LIK_1_5.4	LIK_1_4.6	LIK_1_6.4
LIK_1_4.7	LIK_1_7.4	LIK_1_4.8	LIK_1_8.4	LIK_1_5.6
LIK_1_6.5	LIK_1_5.7	LIK_1_7.5	LIK_1_5.8	LIK_1_8.5
LIK_1_6.7	LIK_1_7.6	LIK_1_6.8	LIK_1_8.6	LIK_1_7.8
LIK_1_8.7	DAS_1_1.2	DAS_1_1.3	DAS_1_1.4	DAS_1_1.5
DAS_1_1.6	DAS_1_1.7	DAS_1_1.8	DAS_1_2.3	DAS_1_2.4
DAS_1_2.5	DAS_1_2.6	DAS_1_2.7	DAS_1_2.8	DAS_1_3.4
DAS_1_3.5	DAS_1_3.6	DAS_1_3.7	DAS_1_3.8	DAS_1_4.5
DAS_1_4.6	DAS_1_4.7	DAS_1_4.8	DAS_1_5.6	DAS_1_5.7
DAS_1_5.8	DAS_1_6.7	DAS_1_6.8	DAS_1_7.8	DM2_1_1.2
DM2_1_1.3	DM2_1_1.4	DM2_1_1.5	DM2_1_1.6	DM2_1_1.7
DM2_1_1.8	DM2_1_2.3	DM2_1_2.4	DM2_1_2.5	DM2_1_2.6
DM2_1_2.7	DM2_1_2.8	DM2_1_3.4	DM2_1_3.5	DM2_1_3.6
DM2_1_3.7	DM2_1_3.8	DM2_1_4.5	DM2_1_4.6	DM2_1_4.7
DM2_1_4.8	DM2_1_5.6	DM2_1_5.7	DM2_1_5.8	DM2_1_6.7
DM2_1_6.8	DM2_1_7.8	AML_1_1.2.3	AML_1_2.1.3	AML_1_3.1.2
AML_1_1.2.4	AML_1_2.1.4	AML_1_4.1.2	AML_1_1.2.5	AML_1_2.1.5
AML_1_5.1.2	AML_1_1.2.6	AML_1_2.1.6	AML_1_6.1.2	AML_1_1.2.7
AML_1_2.1.7	AML_1_7.1.2	AML_1_1.2.8	AML_1_2.1.8	AML_1_8.1.2

AML_1_1.3.4	AML_1_3.1.4	AML_1_4.1.3	AML_1_1.3.5	AML_1_3.1.5
AML_1_5.1.3	AML_1_1.3.6	AML_1_3.1.6	AML_1_6.1.3	AML_1_1.3.7
AML_1_3.1.7	AML_1_7.1.3	AML_1_1.3.8	AML_1_3.1.8	AML_1_8.1.3
AML_1_1.4.5	AML_1_4.1.5	AML_1_5.1.4	AML_1_1.4.6	AML_1_4.1.6
AML_1_6.1.4	AML_1_1.4.7	AML_1_4.1.7	AML_1_7.1.4	AML_1_1.4.8
AML_1_4.1.8	AML_1_8.1.4	AML_1_1.5.6	AML_1_5.1.6	AML_1_6.1.5
AML_1_1.5.7	AML_1_5.1.7	AML_1_7.1.5	AML_1_1.5.8	AML_1_5.1.8
AML_1_8.1.5	AML_1_1.6.7	AML_1_6.1.7	AML_1_7.1.6	AML_1_1.6.8
AML_1_6.1.8	AML_1_8.1.6	AML_1_1.7.8	AML_1_7.1.8	AML_1_8.1.7
AML_1_2.3.4	AML_1_3.2.4	AML_1_4.2.3	AML_1_2.3.5	AML_1_3.2.5
AML_1_5.2.3	AML_1_2.3.6	AML_1_3.2.6	AML_1_6.2.3	AML_1_2.3.7
AML_1_3.2.7	AML_1_7.2.3	AML_1_2.3.8	AML_1_3.2.8	AML_1_8.2.3
AML_1_2.4.5	AML_1_4.2.5	AML_1_5.2.4	AML_1_2.4.6	AML_1_4.2.6
AML_1_6.2.4	AML_1_2.4.7	AML_1_4.2.7	AML_1_7.2.4	AML_1_2.4.8
AML_1_4.2.8	AML_1_8.2.4	AML_1_2.5.6	AML_1_5.2.6	AML_1_6.2.5
AML_1_2.5.7	AML_1_5.2.7	AML_1_7.2.5	AML_1_2.5.8	AML_1_5.2.8
AML_1_8.2.5	AML_1_2.6.7	AML_1_6.2.7	AML_1_7.2.6	AML_1_2.6.8
AML_1_6.2.8	AML_1_8.2.6	AML_1_2.7.8	AML_1_7.2.8	AML_1_8.2.7
AML_1_3.4.5	AML_1_4.3.5	AML_1_5.3.4	AML_1_3.4.6	AML_1_4.3.6
AML_1_6.3.4	AML_1_3.4.7	AML_1_4.3.7	AML_1_7.3.4	AML_1_3.4.8
AML_1_4.3.8	AML_1_8.3.4	AML_1_3.5.6	AML_1_5.3.6	AML_1_6.3.5
AML_1_3.5.7	AML_1_5.3.7	AML_1_7.3.5	AML_1_3.5.8	AML_1_5.3.8
AML_1_8.3.5	AML_1_3.6.7	AML_1_6.3.7	AML_1_7.3.6	AML_1_3.6.8
AML_1_6.3.8	AML_1_8.3.6	AML_1_3.7.8	AML_1_7.3.8	AML_1_8.3.7
AML_1_4.5.6	AML_1_5.4.6	AML_1_6.4.5	AML_1_4.5.7	AML_1_5.4.7
AML_1_7.4.5	AML_1_4.5.8	AML_1_5.4.8	AML_1_8.4.5	AML_1_4.6.7
AML_1_6.4.7	AML_1_7.4.6	AML_1_4.6.8	AML_1_6.4.8	AML_1_8.4.6
AML_1_4.7.8	AML_1_7.4.8	AML_1_8.4.7	AML_1_5.6.7	AML_1_6.5.7
AML_1_7.5.6	AML_1_5.6.8	AML_1_6.5.8	AML_1_8.5.6	AML_1_5.7.8
AML_1_7.5.8	AML_1_8.5.7	AML_1_6.7.8	AML_1_7.6.8	AML_1_8.6.7
